# Polymorphisms in matrix metalloproteinases *MMP1* and *MMP9* are associated with primary open-angle and angle closure glaucoma in a Pakistani population

**Published:** 2013-02-20

**Authors:** Shazia Micheal, Sajeela Yousaf, Muhammad Imran Khan, Farah Akhtar, Farah Islam, Wajid Ali Khan, Anneke I. den Hollander, Raheel Qamar, Asifa Ahmed

**Affiliations:** 1 Department of Biosciences, COMSATS Institute of Information Technology, Islamabad, Pakistan; 2 Department of Ophthalmology, Radboud University Nijmegen Medical Centre; Nijmegen, The Netherlands; 3 Department of Human Genetics, Radboud University Nijmegen Medical Centre, Nijmegen, The Netherlands; 4 Al-Shifa Eye Trust Hospital Jhelum Road, Rawalpindi, Pakistan; 5 Shifa College of Medicine, Shifa Tameer-e-Millat University, Islamabad, Pakistan

## Abstract

**Purpose:**

Matrix metalloproteinases (MMPs) play an important role in remodeling of the extracellular matrix during development and growth of various tissues including the eye. Various functional polymorphisms in MMPs have been implicated in the pathogenesis of different types of glaucoma. The aim of the present study was to investigate the role of various polymorphisms in Pakistani patients with glaucoma.

**Methods:**

The present case-control study included 112 patients with primary open-angle glaucoma (POAG), 82 patients with primary angle closure glaucoma (PACG), and 118 control subjects. Genotyping of polymorphisms was done using PCR followed by restriction fragment length polymorphism analysis.

**Results:**

A significant difference in the genotype frequencies of *MMP1* rs1799750 (−1607 1G/2G) was observed between the patients with POAG and the control subjects (p=0.001). This was attributed to the female subjects (p<0.001), while the association was not significant in male subjects (p>0.47). In addition, a significant difference was observed in genotype frequencies of *MMP9* rs17576 (c.836A>G) in patients with PACG compared to the control subjects (p<0.001), which after gender stratification remained significant in men (p=0.009) but not in women (p=0.14). No significant associations were found for *MMP7* (c.-181T>C) and *MMP9* (c.-1562C>T) polymorphisms.

**Conclusions:**

Our data suggest that the *MMP1* rs1799750 (−1607 1G/2G) and *MMP9* rs17576 polymorphisms might be of value for further study as potential gender-dependent risk factors for developing POAG and PACG, respectively, in Pakistan.

## Introduction

Matrix metalloproteinases (MMPs) are endopeptidases involved in the proteolysis of extracellular matrix (ECM) proteins [1]. The ECM is considered an important determinant for the axial length of the eye. The enhanced activation of collagen degrading enzymes, particularly MMPs, might play a role in the remodeling of the ECM during ocular growth and development. Abnormal expression of MMPs in the eye has been implicated in many disorders, including glaucoma [2,3], proliferative vitreoretinopathy [4-6], cataract formation [7], and pterygia [8,9]. Scholtzer Schrehardt et al. [10] observed that decreased activity of MMPs in the aqueous humor might be involved in the abnormal accumulation of the matrix found in pseudoexfoliation glaucoma (PEXG) and POAG.

Twenty-six MMPs have been discovered, which are grouped into five main classes (collagenases, gelatinases, stromelysins, membrane-type, and others, including matrilysin), based on substrate specificity, homology, and subcellular localization [11]. These MMPs are regulated by activation of latent MMPs and their inhibitors, commonly known as tissue inhibitors of metalloproteinases, but it is believed that regulation at the transcriptional level has a greater impact on the expression of these MMPs. Expression studies revealed that most MMP genes are expressed at the time of tissue remodeling [11]. MMP-1, -2, -3, -9, and -14 have been shown to be expressed in the human sclera.

A cluster of MMP genes (*MMP1*, *MMP3*, *MMP7*, *MMP8*, *MMP10*, *MMP12*, *MMP13*, *MMP20*, *MMP27*) is localized on 11q22. Single nucleotide polymorphisms (SNPs) in the promoter region of various MMPs have been shown to affect transcription levels [12]. Insertion of a G nucleotide at position −1607 in the *MMP1* promoter region has been observed in the core recognition sequence of a transcription factor binding site, which consequently modifies the level of MMP-1 expression [13]. It has been demonstrated that the promoter containing the 2G allele has a significantly higher transcriptional activity than that with the 1G allele [14]. Similarly, a transition from A to G at position −181 in the promoter region of the *MMP7* gene has been reported to result in abnormal activity of MMP-7 [15]. Various functional variants have been identified in the *MMP9* gene, located on 20q11.2-q13.1 [16-18]. The −1562C>T polymorphism in the promoter region exerts a functional effect on gene transcription. Another polymorphism in *MMP9*, 836A>G (rs17576), affects the substrate-binding domain of the MMP-9 enzyme, substituting an uncharged amino acid (glutamine) by a positively charged amino acid (arginine). This polymorphism likely alters the protein conformation, leading to a change in the substrate-binding and enzyme activity [13]. These four SNPs in *MMP1*, *MMP7*, and *MMP9* have been studied in various types of glaucoma in different populations [19,20]. The aim of the current study was to determine whether the polymorphisms in the promoter and coding regions of *MMP1*, *MMP7*, and *MMP9* are associated with POAG and PACG in a Pakistani population.

## Methods

### Patients

All the patients were recruited from the glaucoma clinic of Al-Shifa Trust Eye Hospital in Rawalpindi. Patients were Punjabi in ethnicity (from the Punjab province, located in central Pakistan). The study group consisted of 312 individuals; 112 POAG cases, 82 PACG cases and 118 control subjects. The POAG patients had a mean age of 52.6±1.4 years (69.6% males and 30.4% females), PCAG patients had a mean age of 53.6±1.5 years (males: 48.8%, females 51.2%) and control subjects had a mean age of 50.1±1.3 years (males: 60.2%, females 39.8%). This study conforms to the tenets of the Helsinki declaration and was approved by the Departmental Review and Ethics Committee. All subjects were briefed about the study in their local language, and written informed consent was obtained before their blood samples were obtained. The inclusion criteria for patients and controls, clinical examinations, and collection and processing of blood samples have been described previously [21]. Briefly, for patients with POAG, the inclusion criteria were high intraocular pressure (IOP; >21 mmHg) measured using Goldmann applanation tonometry, a cup-to-disc ratio greater than 0.5, visual field defects typical of glaucoma, which were determined with a Humphrey Field Analyzer (Zeiss Humphery Systems, Dublin, CA), and an open anterior chamber angle. The diagnosis of angle closure was made with gonioscopy, which aids in identifying regions of apposition of the iris to the trabecular meshwork. IOP, cup-disc ratio, and visual field defects criteria for PACG were similar to POAG.

### Genotyping of single nucleotide polymorphisms

Venous blood of patients and healthy individuals was drawn by venipuncture and collected in acid citrate dextrose vacutainers (Becton Dickinson, Franklin Lakes, NJ). Genomic DNA was extracted from whole blood using a standard phenol chloroform method [22] and used for genotyping. Genotypes were determined with PCR–restriction fragment length polymorphism (PCR–RFLP) analysis of the four SNPs studied in *MMP1* rs1799750 (c.-1607–1606insGG), *MMP7* rs11568818 (c.-181T>C), and *MMP9* (rs3918242 c.-1562C>T and rs17576 c.836A>G; p.Gln279Arg). Sequences of the primers used for amplification of the four SNPs are given in Table 1. In the case of *MMP1*, a restriction enzyme site for *Alu*I (AGCT) was introduced in the reverse primer by replacing a T with a G at the penultimate position. The 1G allele has this recognition site, whereas in the 2G allele this site is abolished due to the insertion of an additional guanine [23]. In the *MMP7* reverse primer, a mismatch was introduced at the fourth-to-last base [15]. Annealing temperatures, sizes of PCR products, enzymes used for RFLP, and product sizes obtained after digestion are presented in Table 1 [15,23-25].

**Table 1:** Primers and restriction enzymes used to genotype MMP polymorphisms

SNP	Forward primer	C†	PCR product	R. E	RFLP fragments	Ref
MMP-1=(rs1799750) c.-1607–1606insGG	TGACTTTTAAAACATAGTCTATGTTCA	58	269	*AluI*	1G/1G=241,28 2G/2G=269	[23]
	TCTTGGATTGATTTGAGATAAGTCATAGA					
MMP-7=(rs11568818) c.-181T>C	TGGTACCATAATGTCCTGAATG	65	150	*EcoRI*	T=150 C=120,30	[15]
	TCGTTATTGGCAGGAAGCACACAATGAATT					
MMP-9=(rs3918242) c.-1562C>T	GCCTGGCACATAGTAGGCCC	58	436	*SphI*	C=436 T=242,194	[24]
	CTTCCTAGCCAGCCGGCATC					
MMP-9=(rs17576) c.836A>G	GAGAGATGGGATGAACTG	58	439	*MspI*	A=252,187 G=187, 129, 123	[25]
	GTGGTGGAAATGTGGTGT					

C†=annealing temperature; R.E=Restriction endonuclease

For genotyping of four SNPs *MMP1* rs1799750; *MMP7* rs11568818, *MMP9* (rs3918242 and rs17576), 16 µl aliquot of PCR product was subjected to restriction enzyme digestion at 37° C overnight with 10 U of *Alu*I, EcoRI, *Sph*I, and *Msp*I restriction enzymes, respectively, according to the manufacturer’s instructions (Fermentas, Burlington, Canada). The resulting digested products were resolved on 3% agarose gels (Table 1).

### Statistical analysis

The associations between the genotype and allele frequencies in patients compared to controls were analyzed by computing the Pearson chi-square (χ^2^) and odds ratio (OR 95% confidence interval, CI) using statistical software StatCalc EpiInfo package v.6 (Atlanta, GA). Power analysis was performed with G*Power software version 3.0.8.

## Results

Patients and controls included in the current study were age-matched. The mean age of the controls was 37.9±10.8 years, of patients with POAG 39.5±12.4 years, and of patients with PACG 40.9±16.4 years. In total, 118 healthy subjects (71 men and 47 women), 112 patients with POAG (78 men and 34 women), and 82 patients with PACG patients (40 men and 42 women) were enrolled in the study. The majority of the patients were using medications such as β-blockers to lower IOP. Power calculation indicated that this study had sufficient sample size of controls and cases to detect the previously described effect sizes. Three upstream promoter polymorphisms in *MMP1*, *MMP7*, *MMP9*, and one non-synonymous SNP in *MMP9* were genotyped. Genotype frequencies were consistent with the Hardy–Weinberg equilibrium (HWE) for all four SNPs. A significant difference in genotype frequencies was found for the *MMP1* (rs1799750; c.-1607–1606insGG) SNP in patients with POAG and PACG compared to the controls (Table 2). The homozygous 2G/2G genotype was found at a significantly higher frequency in patients with POAG (OR 3.53 [95% CI=1.63–7.73]; p<0.001) and patients with PACG (OR 2.23 [95% CI=0.96–5.21]; p=0.04). A highly significant association was observed for the 2G allele and patients with POAG (OR 2.04 [95% CI=1.38–3.01); p<0.001]. A weaker association was observed between the 2G allele and patients with PACG (OR 1.61 [95% CI=1.05–2.47]); p=0.02; Table 3).

**Table 2:** *MMP-1* and *MMP-7* SNP genotype frequencies in POAG and PACG patients and unaffected controls

	**Controls** **n=118 (%)**	**POAG** **n=112 (%)**	**p (χ^2^)**	**p (χ^2^)**	**OR (95% CI)**	PACG **n=82 (%)**	**p (χ^2^)**	**p (χ^2^)**	**OR (95% CI)**
*MMP-1*= c.-1607-1606insGG									
**1G/1G**	53(44.9)	27(24)	0.001 (13.04)	Reference		25(30.5)	0.09 (4.75)	Reference	
**1G/2G**	45(38.1)	49(44)		0.01 (5.93)	2.14 (1.10-4.15)	36(43.9)		0.10 (2.58)	1.70 (0.85-3.41)
**2G/2G**	20(17.0)	36(32)		<0.001 (12.35)	3.53 (1.63-7.73)	21(25.6)		0.04 (4.16)	2.23 (0.96-5.21)

*MMP-7* = c.-181T>C									
**TT**	43(36.4)	36(32.1)	0.35 (2.07)	Reference		24(29.3)	0.55 (1.18)	Reference	
**TC**	57(48.3)	64(57.1)		0.31 (1.03)	1.34 (0.73-2.47)	43(52.4)		0.35 (0.86)	1.35 (0.68-2.69)
**CC**	18(15.3)	12(10.8)		0.60 (0.27)	0.80 (0.31-2.03)	15(18.3)		0.35 (0.86)	1.49 (0.59-3.80)

**Table 3:** *MMP-1 *and* MMP-7* SNP genotype frequencies in POAG and PACG patients and unaffected controls

	**Controls n=236 (%)**	**POAG n=224 (%)**	**p (χ^2^)**	**OR (95% CI)**	**PACG** **n=164 (%)**	**p (χ^2^)**	**OR (95% CI)**
*MMP-1= *c.-1607-1606insGG							
**1G**	151(64.0)	103(46)	<0.001 (14.22)	2.04 (1.38-3.01)	86(52.4)	0.02 (4.57)	1.61 (1.05-2.47)
**2G**	85(36.0)	121(54.0)			78(47.6)		

*MMP-7* = c.-181T>C							
**T**	143(60.6)	136(60.7)	0.97 (0.00)	0.99 (0.67-1.47)	91(55.5)	0.30 (1.04)	1.23 (0.81-1.89)
**C**	93(39.4)	88(39.3)			73(44.5)		

A significant association was observed between the GG genotype of the non-synonymous *MMP9* variant (rs17576; c.836A>G; p.Gln279Arg) and patients with PACG (OR 3.73 [95% CI=1.59–8.86]; p<0.001), and to a lesser extent with patients with POAG (OR=2.34 [95% CI=1.09–5.05]; p=0.01; Table 4). Similarly, the G allele was significantly associated with patients with PACG (OR 2.12; [95% CI=1.39–3.26]; p value <0.001) with a higher level of significance compared to patients with POAG (OR 1.60 [95% CI=1.09–2.35]; p=0.01; Table 5).

**Table 4:** *MMP-9 *SNP genotype frequencies in POAG and PACG patients and unaffected controls

	**Controls** **n=118 (%)**	**POAG** **n=112 (%)**	**p (χ^2^)**	**p (χ^2^)**	**OR (95% CI)**	PACG **n=82 (%)**	**p (χ^2^)**	**p (χ^2^)**	**OR (95% CI)**
*MMP-9* = c.-1562C>T									
**CC**	74(62.7)	70 (62.5)	0.24 (2.85)	Reference		56(68.3)	0.23 (2.93)	Reference	
**CT**	37(31.3)	40(35.7)		0.22 (0.63)	1.14 (0.63-2.06)	25(30.5)		0.71 (0.13)	0.89 (0.46-1.73)
**TT**	7(6)	2(1.8)		0.12 (2.37)	0.30 (0.04-1.67)	1(1.2)		0.08 (2.91)	0.19 (0.01-0.60)

*MMP-9* = c.836A>G									
**AA**	40 (33.9)	26(23.2)	0.05 (5.75)	Reference		15(18.3)	<0.001 (12.13)	Reference	
**AG**	53(44.9)	48(42.9)		0.30 (1.07)	1.39 (0.71-2.75)	32(39.0)		0.20 (1.61)	1.61 (0.72-3.60)
**GG**	25(21.2)	38(33.9)		0.01 (5.64)	2.34 (1.09-5.05)	35(42.7)		<0.001 (11.27)	3.73 (1.59-8.86)

**Table 5:** *MMP-9* SNP allele frequencies in POAG and PACG patients and controls

	**Controls n=236 (%)**	**POAG n=224 (%)**	**p (χ^2^)**	**OR (95% CI)**	**PACG** **n=164 (%)**	**p (χ^2^)**	**OR (95% CI)**
*MMP-9* = c.-1562C>T							
**C**	185(78.4)	180(80.4)	0.60 (0.27)	0.89 (0.55-1.43)	137(83.5)	0.20 (1.63)	0.71 (0.41-1.23)
**T**	51(21.6)	44(19.6)			27(16.5)		

*MMP-9* = c.836A>G							
**A**	133(56.4)	100(44.6)	0.01 (6.31)	1.60 (1.09-2.35)	62(37.8)	<0.001 (13.33)	2.12 (1.39-3.26)
**G**	103(43.6)	124(55.4)			102(62.2)		

No significant associations were observed for the *MMP7* (c.-181T>C) and *MMP9* (c.-1562C>T) promoter polymorphisms.

Data were further stratified by gender to study gender-specific associations. The *MMP1* (c.-1607–1606insGG) SNP was found to be significantly associated with POAG (p<0.001, χ^2^=17.20) and PACG in female patients (p=0.03, χ^2^=6.94; Table 6). For *MMP9*, a significant association of the rs17576 SNP was observed with PACG in men (p=0.009, χ^2^=9.21; Table 7).

**Table 6:** *MMP-1 *and *MMP-7 *SNPs genotype frequencies with respect to gender in POAG and PACG patients and unaffected controls

**Males**						**Females**				
	**Controls** **n=71 (%)**	**POAG** **n=78 (%)**	**p (χ^2^)**	PACG **n=40 (%)**	**p (χ^2^)**	**Controls** **n=47 (%)**	**POAG** **n=34 (%)**	**p (χ^2^)**	PACG **n=42 (%)**	**p (χ^2^)**
*MMP-1*= c.-1607-1606insGG										
**1G/1G**	24 (33.8)	20 (25.6)	0.47 (1.48)	10 (25.0)	0.51 (1.34)	29 (61.7)	7 (20.6)	<0.001 (17.20)	15 (35.7)	0.03 (6.94)
**1G/2G**	30 (42.3)	34 (43.6)		17 (42.5)		15 (31.9)	15 (44.1)		19 (45.2)	
**2G/2G**	17 (23.9)	24 (30.8)		13 (32.5)		3 (6.4)	12 (35.3)		8 (19.1)	

*MMP-7* = c.-181T>C										
**TT**	28 (39.4)	22 (28.2)	0.18 (3.33)	9 (22.5)	0.10 (4.47)	15 (31.9)	14 (41.2)	0.68 (0.75)	15 (35.7)	0.68 (0.74)
**TC**	35 (49.3)	50 (64.1)		22 (55.0)		22 (46.8)	14 (41.2)		21 (50.0)	
**CC**	8 (11.3)	6 (7.70)		9 (22.5)		10 (21.3)	6 (17.6)		6 (14.3)	

**Table 7:** *MMP-9* SNPs genotype frequencies with respect to gender in POAG and PACG patients and unaffected controls

**Males**						**Females**				
	**Controls** **n=71 (%)**	**POAG** **n=78 (%)**	**p (χ^2^)**	PACG **n=40 (%)**	**p (χ^2^)**	**Controls** **n=47 (%)**	**POAG** **n=34 (%)**	**p (χ^2^)**	PACG **n=42 (%)**	**p (χ^2^)**
*MMP-9 = *c.-1562C>T										
**CC**	42 (59.2)	49 (62.8)	0.43 (1.68)	26 (65.0)	0.22 (2.97)	32 (68.1)	21 (61.8)	0.32 (2.25)	30 (71.4)	0.86 (0.28)
**CT**	24 (33.8)	27 (34.6)		14 (35.0)		13 (27.7)	13 (38.2)		11 (26.2)	
**TT**	5 (7.0)	2 (2.6)		0 (0.00)		2 (4.2)	0 (0.00)		1 (2.4)	

*MMP-9* = c.836A>G										
**AA**	24 (33.8)	21 (26.9)	0.28 (2.51)	5 (12.5)	0.009 (9.21)	16 (34.0)	5 (14.7)	0.08 (4.88)	10 (23.8)	0.14 (3.86)
**AG**	33 (46.5)	33 (42.3)		18 (45.0)		20 (42.6)	15 (44.1)		14 (33.3)	
**GG**	14 (19.7)	24 (30.8)		17 (42.5)		11 (23.4)	14 (41.2)		18 (42.9)	

## Discussion

In the current study, we detected significant associations of *MMP1* (c.-1607–1606insGG) and *MMP9* polymorphisms (c.836A>G; p.Gln279Arg) with POAG and PACG respectively. Both polymorphisms have been studied previously in patients with POAG and PACG in different populations. A significant association has been described between the homozygous 2G/2G genotype and POAG in a Polish population (OR 1.73; [95% CI=1.05–2.86]; p=0.019) [26]. This is consistent with the current study, although the association detected in Pakistani patients with POAG is stronger (OR 3.53 [95% CI=1.63–7.73]; p<0.001). The current study is the first to describe a significant association of this polymorphism with PACG, but the association is weaker than with POAG.

Previous studies observed significant associations between the *MMP9* polymorphism (rs17576) and PACG in Taiwanese and Australian populations [27,28]. It has been proposed that the short axial length in PACG is perhaps due to an alteration in the activity of MMP-9 in the remodeling of ECM during ocular growth and development.

In a southern Chinese population, no significant association was observed with PACG for rs17576, while a significant association was detected between rs2250889 in *MMP9* and PACG (p=0.004). The *MMP9* rs17576 polymorphism substitutes a positively charged arginine by an uncharged glutamine in a highly conserved gelatinase-specific fibronectin type II domain (FN2), one of three types of internal repeats that combine to form larger domains within fibronectin. This domain in MMP-9 is responsible for the collagen affinity of MMP-9 and presumably enhances substrate binding. These residues might have significant interactions with the surrounding residues, so variations in this amino acid could affect protein stability and function [29-31]. In the present study, a significant association of rs17576 SNP was found with male patients with PACG. Naturally occurring sexual dimorphism has been implicated in the risk and progression of neurodegenerative diseases schizophrenia, Parkinson disease, and Alzheimer disease [32,33]. Various previous studies suggest that these differences between men and women could result from estrogens that downregulate the production and/or release of proinflammatory cytokines such as tumor necrosis factor alpha (TNF-α) [31]. The transcription of MMP genes in turn is enhanced by proinflammatory cytokine TNF-α [34]. This male-specific association could also be reconciled with evidence that women have constitutively a lowered innate immune response [35].

MMP levels in the aqueous humor have previously been found to be significantly raised in patients with POAG and PEXG [36]. Experiments were performed by Ito et al. [37] to determine the effect of antiglaucoma drugs on metabolism within the extracellular matrix of the ocular surface, including the corneal, conjunctival, and subconjunctival areas in the rat. That study suggests that αβ-blockers, α1-blockers, α2-agonists, and prostaglandin derivatives may stimulate ECM degradation of the ocular surface tissue by modulating the balance between MMPs and their inhibitors in the progression of glaucoma, when they are not functioning properly [37]. The precise impact of these polymorphisms on the function of the protein is still unknown, but they could be involved in the partial loss of function of the ECM remodeling during the development and growth of the eye.

In conclusion, our results revealed a significant association of *MMP1* rs1799750 (-1607 1G/2G) and *MMP9* (rs17576) polymorphisms with POAG and PACG, respectively, in a Pakistani population. Additional studies are required to understand the exact role of these polymorphisms in the pathogenesis of glaucoma.
